# Detection of odorants in odour mixtures among healthy people and patients with olfactory dysfunction

**DOI:** 10.1111/ejn.16633

**Published:** 2025-01-13

**Authors:** Eva Drnovsek, Kristina Weitkamp, Venkatesh N. Murthy, Edanur Gurbuz, Antje Haehner, Thomas Hummel

**Affiliations:** ^1^ Smell and Taste Clinic, Department of Otorhinolaryngology Technische Universität Dresden Dresden Germany; ^2^ Center for Brain Science Harvard University Cambridge MA USA; ^3^ Department of Molecular & Cellular Biology Harvard University Cambridge Massachusetts USA; ^4^ Faculty of Medicine Mugla Sitki Kocman University Mugla Turkey

**Keywords:** analytical perception, elemental perception, nose, olfaction, smell

## Abstract

Target odorant detection in mixtures has been shown to become more difficult as the number of background odorants increases and falls below chance level in mixtures with 16 components. Our aim was to investigate target odorant detection in mixtures among healthy people and compare it between dysosmic patients and age‐ and gender‐matched controls. Participants underwent extensive olfactory testing and performed two target odorant detection tasks. Eugenol (‘clove’) and phenylethanol (PEA, ‘rose’) were target odorants for all participants, whereas a third target was randomised. For each target odorant in task one (task two), there were four steps. Mixtures contained two (three) odorants in the first step and up to seven (eight) odorants in the fourth step. In each step, participants were asked to choose the sample with the target odorant from the three (two) jars presented. The study included 90 healthy people and 40 patients. As expected, probability of successful target odorant detection decreased as the number of odorants in the mixture increased. However, even when there were seven (eight) odorants in the mixture, around 50% (50%) of healthy people detected Eugenol and around 30% (40%) detected PEA. Furthermore, both distributions of successful target odorant detection differed from the expected binominal distribution of chance (*p* < 0.001). Patients performed worse at detecting Eugenol or PEA at each step than controls. Furthermore, there were significant positive correlations between task scores and olfactory function. In conclusion, target odorant detection is influenced by the target odorant, number of background odorants, and individual olfactory function.

## Graphical abstract

Humans can detect target odorants in odour mixtures containing up to eight components. Successful target odorant detection depends on the task‐related factors, like the target odorant itself, the number of the background odorants, and on the individual‐related factors such as olfactory function.

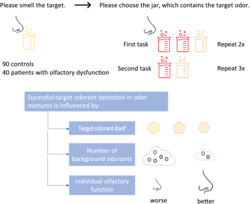

**Abbreviations**
AFC: alternative forced choice
PEA: phenylethanol
TDI: odour threshold (T), odour discrimination (D) odour identification (I)

## 1 INTRODUCTION

Most of the odours we encounter in everyday life are mixtures; however, human perception of mixtures is by far less understood than human perception of single odorants (Thomas‐Danguin et al., 2014). Odour mixtures can be perceived elementally (analytically) or configurally (synthetically). In elemental perception, one perceives its components, while configural perception results in a specific odour, which is different from the individual components (Thomas‐Danguin et al., 2014). Interestingly, it has been shown that some mixtures are perceived configurally among various species (Coureaud et al., 2022). Furthermore, in rats, binary mixtures of similar components were shown to be perceived configurally, while binary mixtures of dissimilar components were perceived elementally (Wiltrout et al., 2003). The latter was also observed in humans (Laing et al., 1984). However, for some mixtures, exactly the opposite was shown (Kay et al., 2005).

In humans, mixtures have been studied mostly in the context of odorant identification; that is, people were asked to identify odorants present in the odour mixture (Laing & Francis, 1989; Laing & Glemarec, 1992; Livermore & Laing, 1998a, 1998b). Noteworthy, they were previously familiarised with the odorants and their names. It has been shown that odorant identification becomes more difficult as the number of odorants increases (Laing & Francis, 1989;Laing & Glemarec, 1992; Livermore & Laing, 1998a, 1998b) and several studies indicated that humans could identify up to three (or at best four) odorants in mixtures. This limit appears to be the same for mixtures of four up to eight components (Laing & Glemarec, 1992; Livermore & Laing, 1998a, 1998b), and it seems to be independent of training, experience (Livermore & Laing, 1996) or odour type (Livermore & Laing, 1998b). Furthermore, odour identification in mixtures has been studied as a potential method to evaluate olfactory function. Liu et al. suggested a Snifffin’ Sticks odour mixture identification test, where two (or three) odours were applied on a piece of paper and participants were asked to identify them. It had good to excellent test–retest reliability and validity for identifying patients with olfactory dysfunction (Liu et al., 2020) indicating that it could be used for evaluation of olfactory function.

Another aspect of odour mixture perception is the ability to detect a target odorant. In other words, participants must decide whether the target odorant is present in a mixture or not. Rokni et al. (2014) showed that mice are easily trained to detect the target odorant in mixtures with even up to 14 compounds. Their accuracy was 94% for one component and declined to 85% when the mixture contained 14 components. On average, each new component reduced their accuracy by 0.75% (Rokni et al., 2014). In a study by Jinks et al., 10 young adults were able to detect a familiar target odorant in mixtures with 12 odorants, whereas detection in mixtures with 16 odorants was below chance level. Therefore, the upper limit for human target odorant detection appears to be between 12 and 16 odorants (Jinks & Laing, 1999).

In this study, we aimed to evaluate human target odorant detection in odour mixtures using two discrimination tasks. More precisely, participants were presented with three or two samples in the first and the second tasks respectively, and they had to choose the one with the target odorant.

Our first aim was to investigate to what extent healthy people can detect a target odorant in odour mixtures and how it changes when increasing the number of odorants. The second aim was to compare the target odorant detection of patients with olfactory dysfunction and age‐ and gender‐matched healthy controls. Furthermore, the test–retest reliability of target odorant detection was evaluated.

## 2 MATERIALS AND METHODS

### 2.1 Participants

People with normal subjective olfactory function (controls) and people with subjective olfactory dysfunctions (patients) were included in the study. An inclusion criterion for both was age of at least 18 years. An inclusion criterion for controls was normal subjective olfactory function and for patients subjective olfactory dysfunction. Exclusion criteria for both were significant health impairments that may interfere with olfactory function such as diabetes mellitus, Parkinson's disease, or renal insufficiency.

Controls were recruited via flyers, while patients were recruited at the Outpatient Clinic of the Smell and Taste Clinic, Department of Otorhinolaryngology, Faculty of Medicine Carl Gustav Carus, Technische Universität Dresden. All participants gave informed written consent. The study was performed in accordance with the Declaration of Helsinki and was approved by the Ethics Committee at the University Clinic of the Technische Universität Dresden (application number EK 15012023).

### 2.2 Olfactory function

Participants filled in a questionnaire regarding socio‐demographic data and subjective olfactory function. Patients underwent a thorough clinical evaluation including a structured medical history. Their olfactory function was evaluated using the extended Sniffin’ Sticks test battery, which yields odour threshold (T), odour discrimination (D) and odour identification (I) scores. The three scores were summed into the TDI score. If the TDI was 30.5 and above, they were diagnosed as normosmic; if the TDI was in the range between 16.5 and 30.25, they were hyposmic; and if the TDI was below 16.5, they were anosmic (Hummel et al., 1997; Oleszkiewicz et al., 2019). Patients also underwent a retronasal odour identification task based on three items (‘taste powders’: Pieniak et al., 2022) and screening of intranasal trigeminal function using an ammonium probe (‘AmmoLa stick’: Sekine et al., 2022). Taste was evaluated using four taste sprays to examine suprathreshold gustatory function and, if clinically relevant, using an extended version with ‘taste strips’ (Landis et al., 2009). Control group's olfactory function was evaluated using only the odour threshold and odour identification from the extended Sniffin’ Sticks battery. If the identification score was above 12, then they were diagnosed as normosmic; if the identification score was between 12 and 8, they were hyposmic; and if the score was below 8, they were anosmic (Oleszkiewicz et al., 2019).

### 2.3 Target odorant detection

**FIGURE 1:**
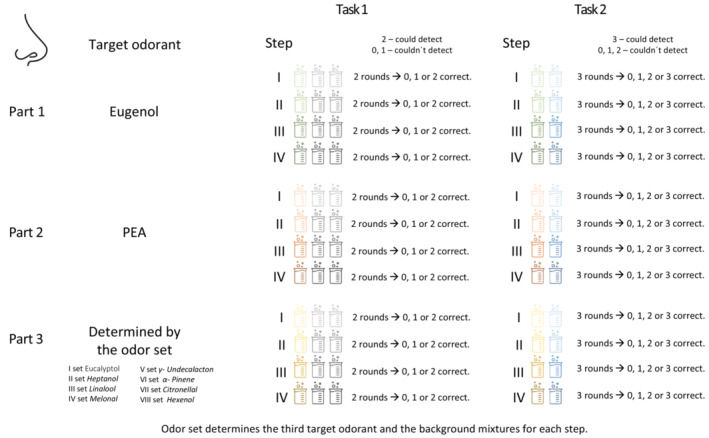
The two tasks for assessment of target odorant detection in odour mixtures. Each task had three parts, one for each target odorant, and each part included four steps (I, II, III, IV). The number of odorants increased with each step. Participants were randomly assigned the odour set, which determined the third target odorant and the composition of the ‘background odorants’ for each step (Table 2).

The target odorant detection in mixtures was evaluated using two tasks (explained in Figure 1; for the odorants see Table 1). Each task had three parts, one for each target odorant. Each part included four steps with increasing number of odorants (I, II, III, IV) (Table 2). Eugenol and PEA were the first and the second target odorant for all participants. Additionally, participants were randomly assigned to one of the eight odour sets, which determined the third target odorant (Undecalacton, Pinene, Melonal, Linalool, Hexenol, Heptanol, Eucalyptol, or Citronellal) and the composition of the ‘background odorants’ for each step (Table 2).

**TABLE 1:** Odorants with their molecular properties, CAS (Chemical Abstract Service reference number), CID (PubChem Compound Identification), perceptual quality, concentration (c), and order numbers from Merck.

Symbol	Trivial name	Abbreviation	CAS	CID	Quality	c	Order numbers
1	Eugenol	Eugenol	97‐53‐0	3314	Cloves	2.5%	W246719
2	2‐Phenylethanol	PEA	60‐12‐8	6054	Rose	5%	77861
A	Eucalyptol	Eucalyptol	470‐82‐6	2758	Eucalyptus	0.5%	C80601
B	2‐Heptanol	Heptanol	534‐49‐7	10976	Mushy, grassy	2%	H3003
C	Linalool	Linalool	78‐70‐6	6549	Bergamot, lavender	2.5%	L260‐2
D	Melonal	Melonal	106‐72‐9	61016	Melon	1%	W238920
E	y‐Undecalacton	Undecalacton	104‐67‐6	7714	Fatty, apricot, peach	4%	W309109
F	a‐Pinene	Pinene	7785‐26‐4	440968	Pine, coniferous tree, resin	2%	147524
G	±‐Citronellal	Citronellal	106‐23‐0	7794	Citrus, lemongrass	1.15%	8.14575
H	Cis‐3‐Hexen‐1‐ol	Hexenol	928‐96‐1	5281167	Grass, freshly cut grass	1.3%	91316

*Note*: Most of the odorants were not 100% pure and included also racemic mixtures. The order numbers are provided for details.

**TABLE 2:** The two target odorant detection tasks. In the first task, there were three jars for each step and each odour set. In the second task, there were two jars for each step and each odour set.

TASK 1												
Set	Step I (two odorants + T)			Step II (three odorants + T)			Step III (five odorants + T)			Step IV (seven odorants + T)		
	T	F	F	T	F	F	T	F	F	T	F	s
**I**	AB + T	AB	AB	ABC + T	ABC	ABC	ABCDE + T	ABCDE	ABCDE	ABCDEFG + T	ABCDEFG	ABCDEFG
**II**	BC + T	BC	BC	BCD + T	BCD	BCD	BCDEF + T	BCDEF	BCDEF	BCDEFGH+ T	BCDEFGH	BCDEFGH
**III**	CD + T	CD	CD	CDE + T	CDE	CDE	CDEFG + T	CDEFG	CDEFG	CDEFGHA + T	CDEFGHA	CDEFGHA
**IV**	DE + T	DE	DE	DEF + T	DEF	DEF	DEFGH + T	DEFGH	DEFGH	DEFGHAB + T	DEFGHAB	DEFGHAB
**V**	EF + T	EF	EF	EFG + T	EFG	EFG	EFGHA + T	EFGHA	EFGHA	EFGHABC + T	EFGHABC	EFGHABC
**VI**	FG + T	FG	FG	FGH + T	FGH	FGH	FGHAB + T	FGHAB	FGHAB	FGHABCD + T	FGHABCD	FGHABCD
**VII**	GH + T	GH	GH	GHA + T	GHA	GHA	GHABC + T	GHABC	GHABC	GHABCDE + T	GHABCDE	GHABCDE
**VIII**	HA + T	HA	HA	HAB + T	HAB	HAB	HABCD + T	HABCD	HABCD	HABCDEF + T	HABCDEF	HABCDEF

TASK 2								
Set	Step I (three odorants)		Step II (four odorants)		Step III (six odorants)		Step IV (eight odorants)	
	T	F	T	F	T	F	T	F
**I**	AB + T	ABC	ABC + T	ABCD	ABCDE + T	ABCDEF	ABCDEFG + T	ABCDEFGH
**II**	BC + T	BCD	BCD + T	BCDE	BCDEF + T	BCDEFG	BCDEFGH + T	BCDEFGHA
**III**	CD + T	CDE	CDE + T	CDEF	CDEFG + T	CDEFGH	CDEFGHA + T	CDEFGHAB
**IV**	DE + T	DEF	DEF + T	DEFG	DEFGH + T	DEFGHA	DEFGHAB + T	DEFGHABC
**V**	EF + T	EFG	EFG + T	EFGH	EFGHA + T	EFGHAB	EFGHABC + T	EFGHABCD
**VI**	FG + T	FGH	FGH + T	FGHA	FGHAB + T	FGHABC	FGHABCD + T	FGHABCDE
**VII**	GH + T	GHA	GHA + T	GHAB	GHABC + T	GHABCD	GHABCDE + T	GHABCDEF
**VIII**	HA + T	HAB	HAB + T	HABC	HABCD + T	HABCDE	HABCDEF + T	HABCDEFG

*Note*: T—true, it includes the target. F—false, it does not include the target. 1 Eugenol, 2 PEA, A Eucalyptol, B Heptanol, C Linalool, D Melonal, E Undecalacton, F Pinene, G Citronellal, H Hexenol, and T Target.

In both tasks, participants were presented with the target odorant at the beginning of each part. They were allowed to smell it for as long as they wanted, and then, they were provided with eye covers for the odour presentation. Each jar was presented 2 cm in front of the nose for approximately 2 s with a 5‐s interval in‐between. After each round, participants were asked to choose the jar with the target odorant in a forced choice manner.

The first was a 3‐alternative forced choice (3‐AFC) task. Two jars contained the ‘background odorants’ without the target odorant, whereas the third contained the mixture with the additional target odorant (Table 2). This task was performed two times in each step.

The second was a 2‐AFC task, where both jars contained the same number of mixture components, but only one of them contained the target odorant (Table 2). This task was performed three times in each step.

At the end, participants were asked whether they had any associations to the target odorant or whether they were able to identify it. Their responses were categorised as ‘unclear association’ (no association or paraphrasing, multiple descriptive words, e.g. fruity, floral, minty) or ‘clear association’ (e.g. cinnamon or cloves for Eugenol).

### 2.4 Odorant selection

Ten pleasant or neutral monomolecular odorants (Eugenol, PEA, Eucalyptol, Heptanol, Linalool, Melonal, Undecalacton, Pinene, Citronellal, and Hexenol) were selected from a previous study by Weiss et al. (2012) (Table 1). Odorants were diluted in propylene glycol to comparable odour intensity as it has been shown that otherwise the more intense odorants predominate (Laing et al., 1984). Initially, odorants were diluted to 2% and 10 participants rated their intensities on a discrete scale from 0 to 10. Eugenol was used as a reference as its intensity ratings were the least variable, with an average of 5.1/10. Three rounds of intensity ratings by 17, 17, and 6 participants were required to achieve an average odour intensity of 4.9. The chosen isointense concentrations are reported in Table 1.

### 2.5 Mixtures preparation

Firstly, individual odorants were diluted in propylene glycol (Table 1). Next, odour mixtures were created (Table 2). Individual odorants were soaked on a gauze pad and put in 50 mL brown glass jars (height 70 mm, diameter of opening 55 mm). The odorants Eugenol and PEA were used only as targets, whereas the remaining eight were used as targets and as background odorants.

### 2.6 Perceptual ratings and trigeminal evaluation of odorants

Additionally, eight healthy people perceptually rated the 10 odorants. There were two appointments; five odorants were evaluated each time. For each odorant, they rated its intensity and irritation on a scale from 0 to 10, pleasantness on a scale from −5 to +5, and whether it was familiar or not. Additionally, to evaluate the importance of the trigeminal component, they performed a target odorant lateralisation task (Frasnelli et al., 2011).

### 2.7 Statistical analysis

The median and interquartile ranges were used to describe the central tendency and variability of continuous variables. Nonparametric Mann–Whitney test was used to compare continuous variables. Frequencies were used to describe the distribution of categorical variables, and *χ* ^2^ test with Monte Carlo simulation (based on 2000 replicates) was used to compare them.

Furthermore, to compare our results to chance, the expected binominal distributions were calculated. In other words, the distributions one would get if all participants chose the ‘different’ sample randomly. The expected binominal distribution for the first (and the second) task assumed that the probability of random success at each trial was 1/3 (and 1/2) and that each person had two (and three) rounds. Next, this expected binominal distribution was compared to our distribution using a *χ* ^2^ test with Monte Carlo simulation (based on 2000 replicates).

In the further analysis, individual scores for each task, each target odorant and each step were interpreted as able to detect the target odorant or not. If all rounds were correct (first task: two out of two rounds; second task: three out of three rounds), then they could detect the target; otherwise, they could not.

Spearman correlation coefficients were used to check for test–retest reliability and to evaluate the correlation between the total scores per task and olfactory function. A *p*‐value of <0.05 was considered significant. All statistical analysis was performed using R Statistical Software (R, 2017) (version 4.2.2; R Foundation for Statistical Computing, Vienna, Austria) with additional packages ‘tidyverse’ (Wickham et al., 2019), and ‘ggpubr’ (Kassambara, 2023).

## 3 RESULTS

This study included 90 people with normal subjective olfactory function (controls) and 40 people with subjective olfactory dysfunction (patients). A subgroup of 40 controls (out of the 90 controls) were age‐ and gender‐matched to the 40 patients. Additionally, 60 participants (51 controls and 9 patients) performed the tasks twice.

### 3.1 Healthy controls

**FIGURE 2:**
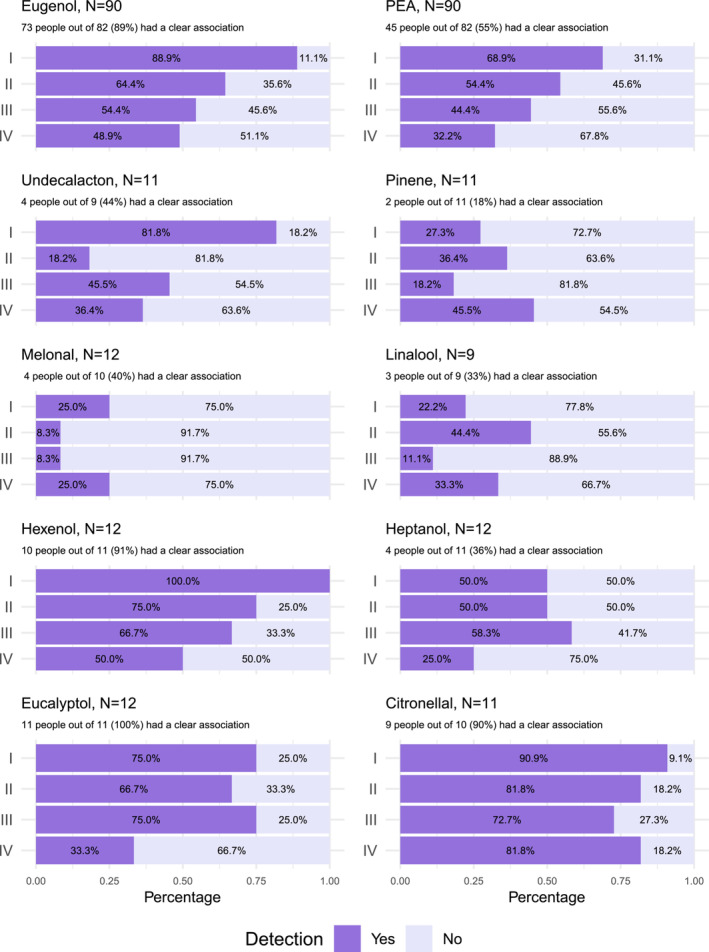
Stacked bar charts of the target odorant detection rates in odour mixtures for the 90 controls for the first task. The dark purple shows those who had 2 out of 2 correct answers (could detect), and the light purple shows those who had 1 or 0 out of 2 correct answers (could not detect). The number of people with a clear association for each target odorant is also reported.

**FIGURE 3:**
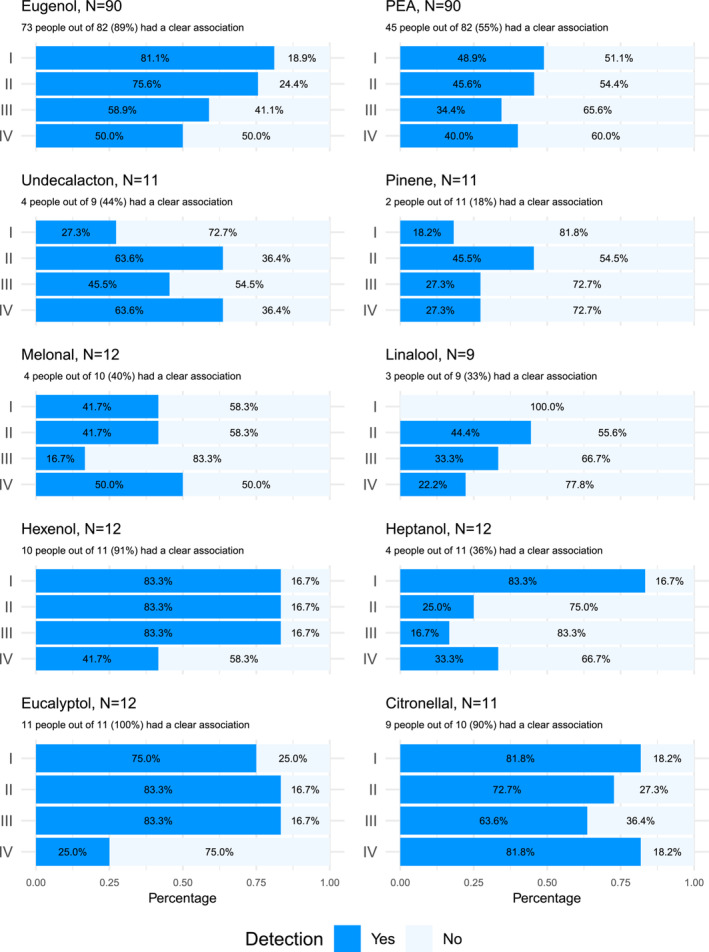
Stacked bar charts of the target odorant detection rates in odour mixtures for the 90 controls for the second task. The dark blue shows those who had 3 out of 3 correct answers (could detect), and the light blue shows those who had 2, 1, or 0 out of 3 correct answers (could not detect). The number of people with a clear association is also reported.

First, target odorant detection was evaluated among 90 healthy people (Table 3). Detection rates were calculated as explained in the methods (first task: 2 out of 2 [could detect], 1 or 0 out of 2 [could not detect]; second task: 3 out of 3 [could detect], 2, 1 or 0 out of 3 [could not detect], for exact scores see Supplement Table S1 and S2) and presented in Figures 2 and 3. Eugenol and PEA detection rates were the highest in the first step and the lowest in the fourth step with the highest number of background odorants. The pattern for other target odorants was not as obvious; however, the sample sizes were also much lower, inviting more variance. Nevertheless, detection rates differed among the target odorants. Interestingly, better odorant recognition was connected to higher detection rates (see also Table 4). Of note, tasks also differed with the composition of the ‘background odorants’; however, further analysis showed that there was no difference in detection rates among the eight odour sets (Supplement Figures S1 and S2). Furthermore, our distributions of correct answers were compared to the expected binominal distribution of chance one would get if participants chose the ‘different’ sample randomly. Both Eugenol and PEA distributions were different from the expected distribution of chance (Supplement Table S1 and S2), indicating people were able to detect the targets.

**TABLE 3:** The sociodemographic data and olfactory function for all the controls (*N* = 90), the subgroup of age‐ and gender‐matched controls (*N* = 40), and patients (*N* = 40).

	Controls (*N* = 90)		Patients (*N* = 40)*	*p* value*
	All the controls (*N* = 90)	Age and gender‐matched (*N* = 40)*		
Age (years)	28.0 (25.0–47.5) [0]	51.5 (41.0–60.5) [0]	54.5 (41.5–61.0) [0]	0.58
Gender (male: female)	29 (32%):61 (68%) [0]	17 (42%):23 (58%) [0]	17 (42%):23 (58%) [0]	1
TDI			25.5 (23.5–28.2) [1]	
Threshold	9.5 (8.3–10.5) [1]	9.8 (8.1–11.1) [1]	3.5 (1.0–6.5) [0]	**< 0.001**
Identification	14.0 (13.0–15.0) [0]	14.0 (12.8–14.0) [0]	11.0 (8.0–12.0) [0]	**< 0.001**
Cause				
COVID‐19			17 (42.5%)	
Viral (non‐COVID19)			5 (12.5%)	
Inflammatory			2 (5%)	
Sinonasal disease			4 (10%)	
Head trauma			1 (2.5%)	
Idiopathic			11 (27.5%)	
Olfactory function**				
Normosmia	74 (82%)	30 (75%)	3 (8%)	
Hyposmia	16 (18%)	10 (25%)	34 (85%)	
Anosmia			2 (5%)	

*Note*: Median (interquartile range) or *N* (%) are reported accordingly. [] indicates the number of people without data.

* *p*‐value compares the age‐ and gender‐matched controls (*N* = 40) and patients' (*N* = 40).

** Based on the 16‐item identification score in controls and based on the TDI in patients.

**TABLE 4:** Total scores for the two tasks (in both tasks there were two parts, the first part with the target odorant Eugenol and the second part with the target odorant PEA) among controls (*N* = 82) in relation to the target odorant recognition (without a clear association and with a clear association).

	Part 1 (Eugenol)			Part 2 (PEA)		
Total score	Unclear association	Clear association	*χ* ^2^ *p* value	Unclear association	Clear association	*χ* ^2^ *p* value
All (*N* = 82)*	9 (11%)	73 (89%)		37 (45%)	45 (55%)
**Task 1**						
**0**	2 (22%)	2 (3%)	***χ*** ^**2**^ **= 10.7** ***p* = 0.03**	5 (14%)	3 (7%)	***χ*** ^**2**^ **= 22.4** ***p* < 0.001**
**1**	2 (22%)	6 (8%)		19 (51%)	7 (15%)	
**2**	3 (33%)	17 (23%)		9 (24%)	9 (20%)	
**3**	1 (11%)	29 (40%)		4 (11%)	18 (40%)	
**4**	1 (11%)	19 (26%)		0 (0%)	8 (18%)	
**Task 2**						
**0**	0 (0%)	2 (3%)	***χ*** ^**2**^ **= 15.7** ***p* = 0.01**	9 (24%)	5 (11%)	***χ*** ^**2**^ **= 14.4** ***p* = 0.004**
**1**	4 (44%)	4 (5%)		18 (49%)	10 (22%)	
**2**	3 (33%)	22 (30%)		7 (19%)	13 (29%)	
**3**	2 (22%)	22 (30%)		2 (5%)	11 (24%)	
**4**	0 (0%)	23 (32%)		1 (3%)	6 (14%)	

* All people who reported, whether they had a clear association with the target odorant or not.

### 3.2 Healthy controls and patients (test validity)

**FIGURE 4:**
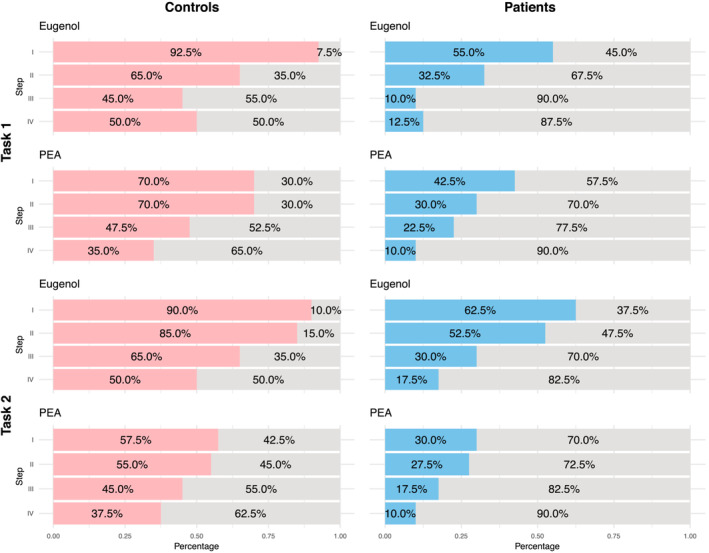
Bar charts of the Eugenol or PEA detection rates among the 40 age‐ and gender‐matched controls (left, in pink) and 40 patients (right, in blue) for the first (upper two graphs) and second task (lower two graphs). Target odorant detection rates are shown for each of the four steps (I, II, III, IV). Pink/blue are the people who could detect the target odorant. The target odorant detection rates per step were significantly lower in patients compared to age‐ and gender‐matched controls (see Table S3–S4). PEA, phenylethanol.

Secondly, target odorant detection of 40 patients and 40 age‐ and gender‐matched controls (Table 3) was compared. Patients had lower Eugenol and PEA detection rates for all four steps in both tasks than age‐ and gender‐matched controls (Figure 4; Supplement Tables S3–S4). Furthermore, the distributions were compared to the expected binominal distribution of chance (Supplement Tables S3 and S4). Interestingly, controls' distributions were different from chance for both target odorants for all four steps. On the other hand, patients' distribution for Eugenol was not different from the expected binominal distribution of chance in the third and the fourth step. Furthermore, patients' distribution for PEA was not different from the expected binominal distribution of chance in the second, the third, and the fourth step in the first task and in all steps in the second task (Supplement Tables S3 and S4). Next, the total scores of Eugenol and PEA detection rates were calculated as the sum of detection rates from 0 (if they could not detect the target in any of the four steps) to 4 (if they could detect the target in all four steps). Patients had lower total Eugenol and PEA detection rates for both tasks compared to the age‐ and gender‐matched controls (Supplement Figure S3).

**FIGURE 5:**
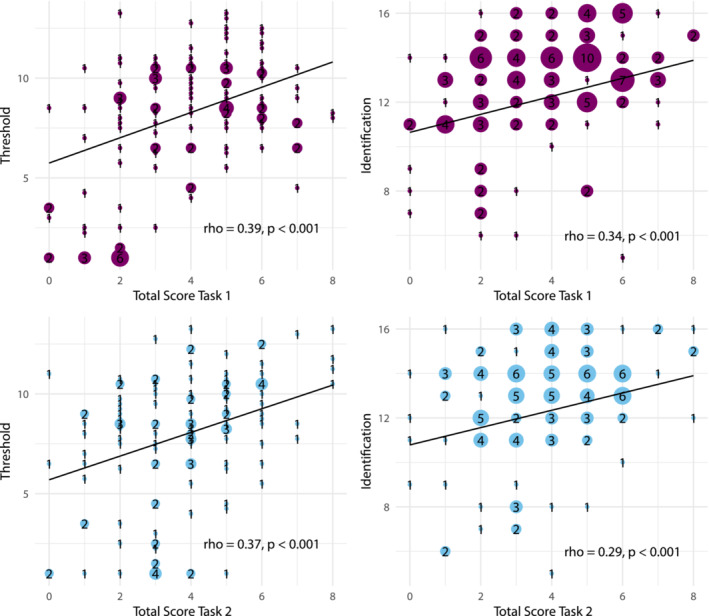
Scatter plots of the total scores for the first and the second task in relation to olfactory function measured using threshold or identification. All controls (*N* = 90) and patients (*N* = 40) are included. The total scores per task for both Eugenol and PEA were calculated as the sum of detection rates from 0 (if they could not detect the target in any of the four steps for both target odorants) to 8 (if they could detect the target in all four steps for both target odorants). PEA, phenylethanol.

Additionally, the relationship between the total detection rates and individual olfactory function was examined among all 130 people (Figure 5). Task scores were correlated with olfactory function (first task total score with odour threshold *ρ* = 0.39, *p* < 0.001 and odour identification *ρ* = 0.34, *p* < 0.001; second task total score with odour threshold *ρ* = 0.37, *p* < 0.001 and odour identification *ρ* = 0.29, *p* < 0.001).

### 3.3 Test – retest reliability

**FIGURE 6:**
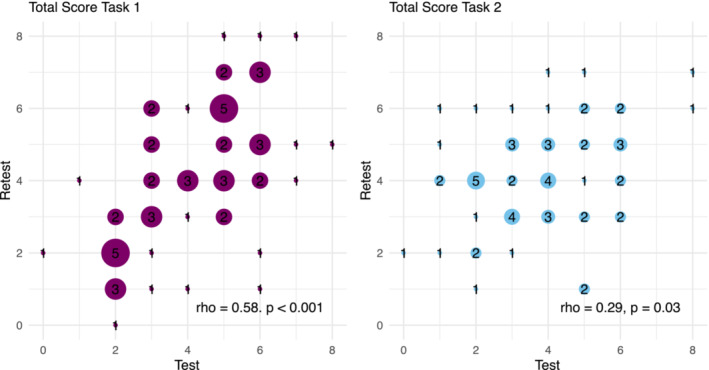
Scatter plots of test–retest reliability among people who underwent the tasks twice (*N* = 60). The total scores per task for both Eugenol and PEA were calculated as the sum of detection rates from 0 (if they could not detect the target in any of the four steps for both target odorants) to 8 (if they could detect the target in all four steps for both target odorants). PEA, phenylethanol.

In the last part, 51 controls and 9 patients performed the tasks twice to check for test–retest reliability. The median (interquartile range) number of days between the first and the second appointments was 21 (7–41) days. Correlation between the first (test) and the second appointments (retest) for the first task was *ρ* = 0.58 (*p* < 0.001) and for the second task *ρ* = 0.29 (*p* = 0.03) as seen in Figure 6.

### 3.4 Perceptual and trigeminal evaluation of the odorants

Additionally, eight people perceptually rated the odorants and performed target odorant lateralisation tasks. In the exploratory analysis, no significant difference in the trigeminal lateralisation scores, pleasantness, intensity, familiarity or irritation ratings among the 10 odorants were observed (Supplement Table S5).

## 4 DISCUSSION

Odorant identification in mixtures is believed to be limited to up to three (or four) odorants (Laing & Francis, 1989; Laing & Glemarec, 1992; Livermore & Laing, 1998a, 1998b), whereas target odorant detection was shown to be possible in mixtures with up to 12 odorants (Jinks & Laing, 1999). Here, we aimed to further investigate to what extent healthy people can detect a single odorant in mixtures and how it differs in patients with olfactory dysfunction. The results indicate that target odorant detection in mixtures is influenced by the target odorant itself, number of background odorants, individual olfactory function and target odorant recognition.

Successful target odorant detection decreased with each step as the number of odorants in mixture increased, which is in line with the previous study of target odorant detection (Jinks & Laing, 1999) and with studies on odorant identification (Laing & Francis, 1989; Laing & Glemarec, 1992; Livermore & Laing, 1998a, 1998b). However, even in the last step, where there were seven odorants in the mixture in the first task and eight odorants in the mixture in the second task, around 50% of healthy people were still successful at detecting Eugenol. Furthermore, around 30% of healthy people in the first task and around 40% in the second task were successful at detecting PEA in the last step. Surprisingly, nine (82%) out of 11 people could still detect Citronellal in the last step (Figures 2 and 3). According to the study by Jinks et al., detection of a familiar odorant remains possible even in mixtures with 12 components and falls below chance in mixtures with 16 odorants (Jinks & Laing, 1999). Unfortunately, this study did not include mixtures with that many components.

While our study showed that humans can analytically perceive mixtures, Weiss et al. (2012) showed that mixtures of isointense odorants spanning the perceptual space become more and more similar to each other as the number of components increases. Even when mixtures with 30 odorants did not share a single component, they were shown to be perceived similarly, which was termed ‘olfactory white’. However, participants could still discriminate between different 30‐component mixtures above chance level (Weiss et al., 2012). Overall, their results support configural perception, whereas our study supports the idea that humans are also well equipped to analytically perceive mixtures with a lower number of components. Of note, our mixtures included only pleasant and neutral odorants as unpleasant odorants can overshadow more pleasant ones (Cain & Drexler, 1974). The mixtures were therefore not covering the whole perceptual space.

In line with Jinks et al., target odorant detection rate varied among the target odorants (Jinks & Laing, 1999). Some odorants such as Citronellal, Hexenol, Eugenol and Eucalyptol were detected much better than others such as Melonal, Linalool and Pinene. This could not be explained by the target odorant perceptual qualities (pleasant, intense, irritating, and familiar) or their trigeminal component as there were no differences among the target odorants at the concentration used in the study (Supplement Table S5). Noteworthy, this analysis included eight people and is only exploratory.

On the other hand, one of the contributing factors for these differences could be target odorant recognition. Target odorants for which more people had a clear association were much better detected. Around 90% of people had a clear association for the odorants with the highest detection rates (Citronellal, Hexenol, Eugenol, and Eucalyptol), whereas only 20%–40% of people had a clear association for the odorants with the lowest detection rates (Melonal, Linalool, and Pinene) (Figures 2 and 3). Noteworthy, the sample sizes for the target odorants, except Eugenol and PEA, were quite small (*N* < 12), and therefore, these observations should be studied further.

Next, the influence of olfactory function on the target odorant detection was examined. Patients with olfactory dysfunction were significantly worse at detecting Eugenol or PEA at each step than age‐ and gender‐matched controls (Figure 4; Supplement Table S3, S4). Additionally, the total task scores were correlated with general olfactory sensitivity (Figure 5), which suggests that a target odorant detection task could be used to evaluate olfactory function in a clinical context. As the test–retest reliability was much better for the first task (Figure 6), it might be better suited for such purposes. Overall, the results support the idea by Liu et al. to use odorant identification in mixtures to evaluate olfactory function (Liu et al., 2020) and extend it to target odorant detection in mixtures.

The present study supports the idea that humans can detect a target odorant in a mixture of up to eight odorants. Recent work in mice using a related experimental strategy appears to indicate that mice are better than humans at this task (Mathis et al., 2016; Rokni et al., 2014). Some reasons for this difference might include the following: (1) Mice are simply better at this task because they have many more odorant receptor types than humans (Hayden et al., 2010; Keller & Vosshall, 2008), and use olfaction a lot in their lives; (2) mice in those experiments were highly trained with reinforcement learning over many days, and humans in the present work were not extensively trained; and (3) the specific choice of odorants and the lack of intensity normalisation in the mouse experiments might have made the task easier for them (however, different target odorants led to similar performances in mice, precluding simple explanations). It is worth noting that theoretical considerations indicate that it is possible to extract the identity of the target odorant in large mixtures from the combined activity of the receptor array, as long as there are a large number of odorant receptor types and the odour tuning of each receptor type is relatively sparse (Mathis et al., 2016). In fact, mutant mice with even a small fraction of the olfactory bulb remaining can perform this target detection task surprisingly well (Licht et al., 2023). Therefore, it is likely that brains have evolved to perform this computation robustly, as it may be important for survival.

One potential limitation of this study is that most of the odorants were not 100% pure and included also racemic mixtures. Another limitation is that the four steps within each part were performed in the same order, from the first with the lowest number of background odorants to the fourth with the highest number of background odorants, whereas the target odorant was presented only at the beginning of each part of the task. Therefore, the time from the presentation to the performance was always the longest in the fourth step. In follow‐up studies, we would suggest randomising the steps as well. Furthermore, it would be interesting to include mixtures with up to 16 components to better determine the limit for target odorant detection (Jinks & Laing, 1999).

## 5 CONCLUSION

This study confirmed that healthy people can detect the target odorants in mixtures with up to eight components above chance level. Overall, human target odorant detection was influenced by the target odorant, number of background odorants, individual olfactory function, and target odorant recognition.

## AUTHOR CONTRIBUTIONS

ED and KW contributed equally. KW, VNM, AH and TH designed the research. KW and EG performed the research. ED analyzed data. ED and TH wrote the paper. All authors revised the paper.

## CONFLICT OF INTEREST STATEMENT

Since 2021, TH has collaborated on research projects with Sony, Stuttgart, Germany; Smell and Taste Lab, Geneva, Switzerland; Takasago, Paris, France; and aspuraclip, Berlin, Germany. He received consultancy fees from Baia Foods, Madrid, Spain; Burghart, Holm, Germany; and air‐up, Munich, Germany. Other authors have no conflict of interest to declare.

### PEER REVIEW

The peer review history for this article is available at https://www.webofscience.com/api/gateway/wos/peer-review/10.1111/ejn.16633.

## Supporting information


**Table S1.** Number of correct answers for each target odorant in the first task among controls (N = 90). The expected distribution of chance (if all people randomly chose the “different” sample) is reported and compared to the observed distribution.
**Table S2.** Number of correct answers for the target odorants in the second task among controls (N = 90). The expected distribution of chance (if all people randomly chose the “different” sample) is reported and compared to the observed distribution.
**Table S3.** Number of correct answers for the first task among the 40 age‐ and gender‐matched controls and 40 patients with olfactory dysfunction for Eugenol and PEA per step. Expected distribution of chance (if all people randomly chose the “different” sample) is reported and compared to the observed distribution.
**Table S4.** Number of correct answers for the second task among the 40 age‐ and gender‐matched controls and 40 patients with olfactory dysfunction for Eugenol and PEA per step. Expected distribution of chance (if all people randomly chose the “different” sample) is reported and compared to the observed distribution.
**Table S5.** Odorants used in the study with their perceptual descriptor ratings (pleasant, intensity, familiar, irritating) and the trigeminal lateralization score among healthy people (N = 8).
**Figure S1.** Eugenol and PEA detection ratesfor the controls (N = 90) forthe first task. Each graph shows the detection rates for one step for the eight odor sets.
**Figure S2.** Eugenol and PEA detection rates bar charts for the controls (N = 90) for the second task. Each graph shows the detection rates for one step for the eight odor sets.
**Figure S3.** Histograms of the Eugenol detection, PEA detection, and total task scores per task for ageand gender‐matched controls (N = 40, above, in pink) and patients (N = 40, below, in blue). Scores were calculated as a sum of successful detection rates for the target odorant for the four steps.

## Data Availability

Code and data are available upon request on: https://doi.org/10.7303/syn53277070.
