# Supplementary material for: Detection of odorants in odour mixtures among healthy people and patients with olfactory dysfunction
Source: Eur J Neurosci. 2025 Jan 13;61(1):e16633. doi: 10.1111/ejn.16633 (PMC11727005; doi:10.1111/ejn.16633)
Supplement: Supplementary file 1 — Table S1. Number of correct answers for each target odorant in the first task among controls (N = 90). The expected distribution of chance (if all people randomly chose the “different” sample) is reported and compared to the observed distribution. Table S2. Number of correct answers for the target odorants in the second task among controls (N = 90). The expected distribution of chance (if all people randomly chose the “different” sample) is reported and compared to the observed distribution. Table S3. Number of correct answers for the first task among the 40 age‐ and gender‐matched controls and 40 patients with olfactory dysfunction for Eugenol and PEA per step. Expected distribution of chance (if all people randomly chose the “different” sample) is reported and compared to the observed distribution. Table S4. Number of correct answers for the second task among the 40 age‐ and gender‐matched controls and 40 patients with olfactory dysfunction for Eugenol and PEA per step. Expected distribution of chance (if all people randomly chose the “different” sample) is reported and compared to the observed distribution. Table S5. Odorants used in the study with their perceptual descriptor ratings (pleasant, intensity, familiar, irritating) and the trigeminal lateralization score among healthy people (N = 8). Figure S1. Eugenol and PEA detection ratesfor the controls (N = 90) forthe first task. Each graph shows the detection rates for one step for the eight odor sets. Figure S2. Eugenol and PEA detection rates bar charts for the controls (N = 90) for the second task. Each graph shows the detection rates for one step for the eight odor sets. Figure S3. Histograms of the Eugenol detection, PEA detection, and total task scores per task for ageand gender‐matched controls (N = 40, above, in pink) and patients (N = 40, below, in blue). Scores were calculated as a sum of successful detection rates for the target odorant for the four steps. [file EJN-61-0-s001.pdf]

SUPPLEMENT

Detection of odorants in odor mixtures among healthy people and patients with olfactory dysfunction

Running title: Detection of odorants in mixtures

Eva Drnovsek*[1], Kristina Weitkamp*[1], Venkatesh N. Murthy[2,3], Edanur Gurbuz[1,4], Antje Haehner[1], Thomas Hummel[1]

*Contributed equally

[1]Smell and Taste Clinic, Department of Otorhinolaryngology, Technische Universität Dresden, Dresden, Germany

[2]Center for Brain Science, Harvard University, Cambridge, MA 02138

[3]Department of Molecular & Cellular Biology, Harvard University, Cambridge, MA 02138

[4]Faculty of Medicine, Mugla Sitki Kocman University, Mugla, Turkye

Table S1. Number of correct answers for each target odorant in the first task among controls (N = 90). The expected distribution of chance (if all people randomly chose the "different" sample) is reported and compared to the observed distribution.

Task 1

	**0/2 correct**	**1/2 correct**	**2/2 correct**	**χ^{2}***	**p value***
**Eugenol (N=90)**					
**I**	1 (1%)	9 (10%)	80 (89%)	**111.2**	**< 0.001**
**II**	2 (2%)	30 (33%)	58 (65%)	**69.7**	**< 0.001**
**III**	9 (10%)	32 (36%)	49 (54%)	**46.3**	**< 0.001**
**IV**	9 (10%)	37 (41%)	44 (49%)	**41.1**	**< 0.001**
**Expected distribution**	40 (44.4%)	40 (44.4%)	10 (11.1%)		
**PEA (N=90)**					
**I**	8 (9%)	20 (22%)	62 (69%)	**65.6**	**< 0.001**
**II**	14 (16%)	27 (30%)	49 (54%)	**40.8**	**< 0.001**
**III**	15 (17%)	35 (39%)	40 (44%)	**29.7**	**< 0.001**
**IV**	19 (21%)	42 (47%)	29 (32%)	**16.8**	**< 0.001**
**Expected distribution**	40 (44.4%)	40 (44.4%)	10 (11.1%)		
**Undecalacton (N=11)**					
**I**	0 (0%)	2 (18%)	9 (82%)	**12.1**	**< 0.001**
**II**	3 (27%)	6 (55%)	2 (18%)	0.75	0.83
**III**	2 (18%)	4 (36%)	5 (46%)	3.6	0.14
**IV**	3 (27%)	4 (36%)	4 (36%)	2.0	0.46
**Expected distribution**	4.9 (44.4%)	4.9 (44.4%)	1.2 (11.1%)		
**Pinene (N=11)**					
**I**	3 (27%)	5 (46%)	3 (27%)	1.2	0.83
**II**	1 (9%)	6 (55%)	4 (36%)	4.2	0.12
**III**	4 (36%)	5 (46%)	2 (18%)	0.28	1.00
**IV**	1 (9%)	5 (46%)	5 (46%)	4.9	0.09
**Expected distribution**	4.9 (44.4%)	4.9 (44.4%)	1.2 (11.1%)		
**Melonal (N=12)**					
**I**	4 (33%)	5 (42%)	3 (25%)	0.84	0.73
**II**	4 (33%)	7 (58%)	1 (8%)	0.46	0.82
**III**	6 (50%)	5 (42%)	1 (8%)	0.10	1.00
**IV**	3 (25%)	6 (50%)	3 (25%)	1.3	0.55
**Expected distribution**	5.3 (44.4%)	5.3 (44.4%)	1.3 (11.1%)		
**Linalool (N=9)**					
**I**	3 (33%)	4 (44%)	2 (22%)	0.48	1.00
**II**	1 (11%)	4 (44%)	4 (44%)	3.6	0.17
**III**	6 (67%)	2 (22%)	1 (11%)	1.1	1.00

**IV**	3 (33%)	3 (33%)	3 (33%)	1.3	0.69
**Expected distribution**	4 (44.4%)	4 (44.4%)	1 (11.1%)		
**Hexenol (N=12)**					
**I**	0 (0%)	0 (0%)	12 (100%)	**19.2**	**< 0.001**
**II**	1 (8%)	2 (17%)	9 (75%)	**10.2**	**0.004**
**III**	2 (17%)	2 (17%)	8 (67%)	**7.8**	**0.01**
**IV**	0 (0%)	6 (50%)	6 (50%)	**8.3**	**0.01**
**Expected distribution**	5.3 (44.4%)	5.3 (44.4%)	1.3 (11.1%)		
**Heptanol (N=12)**					
**I**	2 (17%)	4 (33%)	6 (50%)	4.7	0.12
**II**	2 (17%)	4 (33%)	6 (50%)	4.7	0.12
**III**	2 (17%)	3 (25%)	7 (58%)	**6.0**	**0.049**
**IV**	4 (33%)	5 (42%)	3 (25%)	0.84	0.74
**Expected distribution**	5.3 (44.4%)	5.3 (44.4%)	1.3 (11.1%)		
**Eucalyptol (N=12)**					
**I**	0 (0%)	3 (25%)	9 (75%)	**11.7**	**< 0.001**
**II**	0 (0%)	4 (33%)	8 (67%)	**10.3**	**0.003**
**III**	1 (9%)	2 (17%)	9 (75%)	**10.1**	**0.004**
**IV**	5 (42%)	3 (25%)	4 (33%)	2.0	0.48
**Expected distribution**	5.3 (44.4%)	5.3 (44.4%)	1.3 (11.1%)		
**Citronellal (N=12)**					
**I**	0 (0%)	1 (9%)	10 (91%)	**14.9**	**< 0.001**
**II**	2 (18%)	0 (0%)	9 (82%)	**12.5**	**0.001**
**III**	1 (9%)	2 (18%)	8 (73%)	**9.2**	**0.01**
**IV**	1 (9%)	1 (9%)	9 (82%)	**11.6**	**< 0.001**
**Expected distribution**	5.3 (44.4%)	5.3 (44.4%)	1.3 (11.1%)		

*Chi-squared test with Monte Carlo simulation is reported.

Table S2. Number of correct answers for the target odorants in the second task among controls (N=90). The expected distribution of chance (if all people randomly chose the "different" sample) is reported and compared to the observed distribution.

	Task 2					
	**0/3 correct**	**1/3 correct**	**2/3 correct**	**3/3 correct**	**χ^{2}***	**p value***
**Eugenol (N=90)**						
**I**	0 (0%)	4 (4%)	13 (14%)	73 (81%)	**89.2**	**<0.001**
**II**	1 (1%)	5 (6%)	16 (18%)	68 (76%)	**76.9**	**<0.001**
**III**	2 (2%)	8 (9%)	27 (30%)	53 (59%)	**50.2**	**<0.001**
**IV**	3 (3%)	15 (17%)	27 (30%)	45 (50%)	**32.9**	**<0.001**
**Expected distribution**	11.3 (12.5%)	33.8 (37.5%)	33.8 (37.5%)	11.3 (12.5%)		
**PEA (N=90)**						
**I**	7 (8%)	16 (18%)	23 (26%)	44 (49%)	**28.8**	**<0.001**
**II**	5 (6%)	12 (13%)	32 (36%)	41 (46%)	**29.7**	**<0.001**
**III**	11 (12%)	10 (11%)	38 (42%)	31 (34%)	**22.4**	**<0.001**
**IV**	6 (7%)	17 (19%)	31 (34%)	36 (40%)	**20.2**	**<0.001**
**Expected distribution**	11.3 (12.5%)	33.8 (37.5%)	33.8 (37.5%)	11.3 (12.5%)		
**Undecalacton (N=11)**						
**I**	3 (27%)	2 (18%)	3 (27%)	3 (27%)	2.1	0.56
**II**	0 (0%)	1 (9%)	3 (27%)	7 (64%)	**7.2**	**0.04**
**III**	0 (0%)	3 (27%)	3 (27%)	5 (46%)	3.8	0.25
**IV**	0 (0%)	1 (9%)	3 (27%)	7 (64%)	**7.2**	**0.04**
**Expected distribution**	1.4 (12.5%)	4.1 (37%)	4.1 (37%)	1.4 (12.5%)		
**Pinene (N=11)**						
**I**	2 (18%)	3 (27%)	4 (36%)	2 (18%)	0.41	1.00
**II**	1 (9%)	3 (27%)	2 (18%)	5 (46%)	3.0	0.44
**III**	0 (0%)	5 (45%)	3 (27%)	3 (27%)	2.2	0.50
**IV**	1 (9%)	3 (27%)	4 (36%)	3 (27%)	0.84	0.85
**Expected distribution**	1.4 (12.5%)	4.1 (37%)	4.1 (37%)	1.4 (12.5%)		
**Melonal (N=12)**						
**I**	2 (17%)	2 (17%)	3 (25%)	5 (42%)	3.2	0.43
**II**	0 (0%)	3 (25%)	4 (33%)	5 (42%)	3.7	0.27
**III**	1 (8%)	4 (33%)	5 (42%)	2 (17%)	0.22	1.00
**IV**	2 (17%)	1 (8%)	3 (25%)	6 (50%)	5.3	0.17
**Expected distribution**	1.5 (12.5%)	4.5 (37.5%)	4.5 (37.5%)	1.5 (12.5%)		
**Linalool (N=9)**						
**I**	0 (0%)	3 (33%)	6 (67%)	0 (0%)	3.0	0.31
**II**	2 (22%)	2 (22%)	1 (11%)	4 (44%)	3.5	0.35

**III**	1 (11%)	4 (44%)	1 (11%)	3 (33%)	2.2	0.59
**IV**	1 (11%)	3 (33%)	3 (33%)	2 (22%)	0.30	1.00
**Expected distribution**	1.1 (12.5%)	3.4 (37.5%)	3.4 (37.5%)	1.1 (12.5%)		
**Hexenol (N=12)**						
**I**	0 (0%)	0 (0%)	2 (17%)	10 (83%)	**13.3**	**< 0.001**
**II**	1 (8%)	0 (0%)	1 (8%)	10 (83%)	**13.1**	**< 0.001**
**III**	1 (8%)	0 (0%)	1 (8%)	10 (83%)	**13.1**	**< 0.001**
**IV**	0 (0%)	1 (8%)	6 (50%)	5 (42%)	5.8	0.10
**Expected distribution**	1.5 (12.5%)	4.5 (37.5%)	4.5 (37.5%)	1.5 (12.5%)		
**Heptanol (N=12)**						
**I**	1 (8%)	0 (0%)	1 (8%)	10 (83%)	**13.1**	**< 0.001**
**II**	1 (8%)	2 (17%)	6 (50%)	3 (25%)	1.8	0.78
**III**	0 (0%)	4 (33%)	6 (50%)	2 (17%)	1.8	0.66
**IV**	0 (0%)	3 (25%)	5 (42%)	4 (33%)	2.9	0.38
**Expected distribution**	1.5 (12.5%)	4.5 (37.5%)	4.5 (37.5%)	1.5 (12.5%)		
**Eucalyptol (N=12)**						
**I**	0 (0%)	1 (8%)	2 (17%)	9 (75%)	**10.1**	**0.006**
**II**	0 (0%)	1 (8%)	1 (8%)	10 (83%)	**12.2**	**0.001**
**III**	0 (0%)	0 (0%)	2 (17%)	10 (83%)	**13.3**	**< 0.001**
**IV**	1 (8%)	4 (33%)	4 (33%)	3 (25%)	0.66	1.00
**Expected distribution**	1.5 (12.5%)	4.5 (37.5%)	4.5 (37.5%)	1.5 (12.5%)		
**Citronellal (N=12)**						
**I**	0 (0%)	0 (0%)	2 (18%)	9 (81%)	**11.8**	**0.002**
**II**	1 (9%)	1 (9%)	1 (9%)	8 (73%)	**8.6**	**0.02**
**III**	1 (9%)	1 (9%)	2 (18%)	7 (64%)	6.5	0.08
**IV**	1 (9%)	0 (0%)	1 (9%)	9 (82%)	**11.7**	**0.003**
**Expected distribution**	1.5 (12.5%)	4.5 (37.5%)	4.5 (37.5%)	1.5 (12.5%)		

*Chi-squared test with Monte Carlo simulation is reported.

Table S3. Number of correct answers for the first task among the 40 age- and gender-matched controls and 40 patients with olfactory dysfunction for Eugenol and PEA per step. Expected distribution of chance (if all people randomly chose the “different” sample) is reported and compared to the observed distribution.

Task 1

	Controls (N=40)					Patients (N=40)					Comp. patients and controls*	
	0/2 correct	1/2 correct	2/2 correct	χ^2	p**	0/2 correct	1/2 correct	2/2 correct	χ^2	p**	χ^{2*}	p*
**Eugenol**												
I	0 (0%)	3 (8%)	37 (93%)	**53.9**	**<0.001**	3 (8%)	15 (37.5%)	22 (55%)	**22.4**	**<0.001**	**14.5**	**0.005**
II	1 (2.5%)	13 (32.5%)	26 (65%)	**31.0**	**<0.001**	10 (25%)	17 (42.5%)	13 (32.5%)	**6.4**	**0.04**	**8.5**	**0.01**
III	6 (15%)	16 (40%)	18 (45%)	**14.1**	**0.001**	13 (32.5%)	23 (57.5%)	4 (10%)	1.4	0.58	**12.3**	**0.006**
IV	2 (5%)	18 (45%)	20 (50%)	**22.5**	**<0.001**	10 (25%)	25 (62.5%)	5 (12.5%)	3.4	0.19	**13.1**	**0.006**
**PEA**												
I	2 (5%)	10 (25%)	28 (70%)	**31.9**	**<0.001**	11 (27.5%)	12 (30%)	17 (42.5%)	**10.1**	**0.008**	**6.1**	**0.03**
II	5 (12.5%)	7 (17.5%)	28 (70%)	**29.0**	**<0.001**	14 (35%)	14 (35%)	12 (30%)	4.4	0.11	**12.8**	**0.006**
III	4 (10%)	17 (42.5%)	19 (47.5%)	**17.8**	**<0.001**	10 (25%)	21 (52.5%)	9 (22.5%)	4.0	0.15	**5.5**	**0.03**
IV	11 (27.5%)	15 (37.5%)	14 (35%)	**6.8**	**0.04**	20 (50%)	16 (40%)	4 (10%)	0.25	0.94	**7.2**	**0.02**
**Expected distribution**	17.8 (44.4%)	17.8 (44.4%)	4.4 (11.1%)			17.8 (44.4%)	17.8 (44.4%)	4.4 (11.1%)				

Footnote:

*Detection rates (2 out of 2 [could detect], 1 or 0 out of 2 [could not detect]) are compared between patients and age- and gender-matched controls. P values with Benjamini-Hochkberg adjustment for multiple testing are reported.
**Expected distribution of chance is compared to our distribution using a chi-squared test with Monte Carlo simulation.

Table S4. Number of correct answers for the second task among the 40 age- and gender-matched controls and 40 patients with olfactory dysfunction for Eugenol and PEA per step. Expected distribution of chance (if all people randomly chose the "different" sample) is reported and compared to the observed distribution.

Task 2														
	**Controls (N=40)**						**Patients (N=40)**						**Comp. patients and controls***	
	**0/3 correct**	**1/3 correct**	**2/3 correct**	**3/3 correct**	**χ^2**	**p****	**0/3 correct**	**1/3 correct**	**2/3 correct**	**3/3 correct**	**χ^2**	**p****	**χ^{2}***	**p***
**Eugenol**														
**I**	0 (0%)	1 (2.5%)	3 (7.5%)	36 (90%)	**48.7**	**< 0.001**	1 (2.5%)	2 (5%)	12 (30%)	25 (62.5%)	**26.3**	**< 0.001**	**8.4**	**0.01**
**II**	0 (0%)	1 (2.5%)	5 (12.5%)	34 (85%)	**43.8**	**< 0.001**	2 (5%)	5 (10%)	12 (30%)	21 (52.5%)	**16.5**	**< 0.001**	**9.8**	**0.01**
**III**	0 (0%)	3 (7.5%)	11 (27.5%)	26 (65%)	**27.8**	**< 0.001**	3 (7.5%)	9 (22.5%)	16 (40%)	12 (30%)	4.9	0.21	**9.8**	**0.01**
**IV**	2 (5%)	7 (17.5%)	11 (27.5%)	20 (50%)	**13.8**	**0.003**	4 (10%)	14 (35%)	15 (37.5%)	7 (17.5%)	0.5	0.95	**9.4**	**0.01**
**PEA**														
**I**	1 (2.5%)	9 (22.5%)	7 (17.5%)	23 (57.5%)	**18.6**	**< 0.001**	3 (7.5%)	10 (25%)	25 (37.5%)	12 (30%)	4.4	0.25	**6.1**	**0.03**
**II**	0 (0%)	5 (12.5%)	13 (32.5%)	22 (55%)	**20.9**	**< 0.001**	5 (12.5%)	12 (30%)	12 (30%)	11 (27.5%)	2.9	0.43	**6.2**	**0.03**
**III**	1 (2.5%)	5 (12.5%)	16 (40%)	18 (45%)	**15.1**	**0.002**	8 (20%)	13 (32.5%)	12 (30%)	7 (17.5%)	1.5	0.71	**7.0**	**0.02**
**IV**	1 (2.5%)	10 (25%)	14 (35%)	15 (37.5%)	**8.7**	**0.03**	6 (15%)	16 (40%)	14 (35%)	4 (10%)	0.3	1.00	**8.4**	**0.01**
**Expected distribution**	5 (12.5%)	15 (37.5%)	15 (37.5%)	5 (12.5%)			5 (12.5%)	15 (37.5%)	15 (37.5%)	5 (12.5%)				

Footnote:

*Detection rates (3 out of 3 [could detect], 2, 1 or 0 out of 3 [could not detect]) are compared between patients and age- and gender-matched controls. P values with Benjamini-Hochkberg adjustment for multiple testing are reported.
**Expected distribution of chance is compared to our distribution using a chi-squared test with Monte Carlo simulation.

Table S5. Odorants used in the study with their perceptual descriptor ratings (pleasant, intensity, familiar, irritating) and the trigeminal lateralization score among healthy people (N = 8).

Odorant	Pleasant**	Intensity***	Irritating***	Familiar^	Lateralization^^
**Eugenol**	1.0 (-1 – 3)	4.5 (3.8 – 5.5)	0.5 (0 – 1.8)	5 (63%)	8.0 (6.0 – 10.1)
**PEA**	2.0 (0.8 – 4.0)	3.5 (2.0 – 5.0)	0.0 (0.0 – 0.0)	4 (50%)	11.5 (8.3 – 14.0)
**Eucalyptol**	2.5 (2.0 – 3.0)	5.5 (3.0 – 7.0)	0.0 (0.0 – 0.0)	8 (100%)	12.0 (8.0 – 15.8)
**Heptanol**	1.0 (-0.3 – 3.0)	5.0 (3.8 – 7.0)	0.0 (0.0 – 3.0)	4 (50%)	12.0 (9.8 - 17.0)
**Linalool**	3.5 (0.3 – 4.3)	4.5 (3.8 – 6.3)	0.0 (0.0 – 1.5)	7 (88%)	11.0 (7.75 – 11.3)
**Melonal**	3.0 (1.5 – 4.3)	6.5 (5.5 – 7.3)	0.0 (0.0 – 0.5)	7 (88%)	11.0 (10.0 – 13.8)
**Undecalacton**	3.0 (1.5 – 4.3)	4.0 (3.8 – 5.5)	0.0 (0.0 – 0.0)	6 (75%)	12.5 (10.8 – 14.5)
**Pinene**	1.0 (-0.8 – 2.5)	3.5 (3.0 – 4.0)	0.0 (0.0 – 1.8)	4 (50%)	11.0 (8.5 – 15.0)
**Citronellal**	2 (-0.3 – 4)	7.0 (4.8 – 8.0)	0.0 (0.0 -2.0)	8 (100%)	11.5(8.8 – 13.5)
**Hexenol**	1.5 (0.0 – 3.3)	5.0 (3.8 – 6.3)	0.0 (0.0 -0.3)	7 (88%)	11.5 (10.0 – 17.0)
*Comparison**	*χ2(9) = 6.1, P = 0.74*	*χ2(9) = 12.5, P = 0.19*	*χ2(9) = 8.9, P = 0.45*	*χ2 = 16, P = 0.07*	*χ2(9) = 7.4, P = 0.59*

Footnote:

*Kruskal Wallis or χ^2tests were used accordingly.

**on a discrete scale from -5 to 5

***on a discrete scale from 0 to 10

^Two options: familiar or not. Percentage of people who found the odorant familiar is reported.

^^Lateralization task was repeated 20 times, and the number of correct answers is reported.

Figure S1. Eugenol and PEA detection rates for the controls (N = 90) for the first task. Each graph shows the detection rates for one step for the eight odor sets.

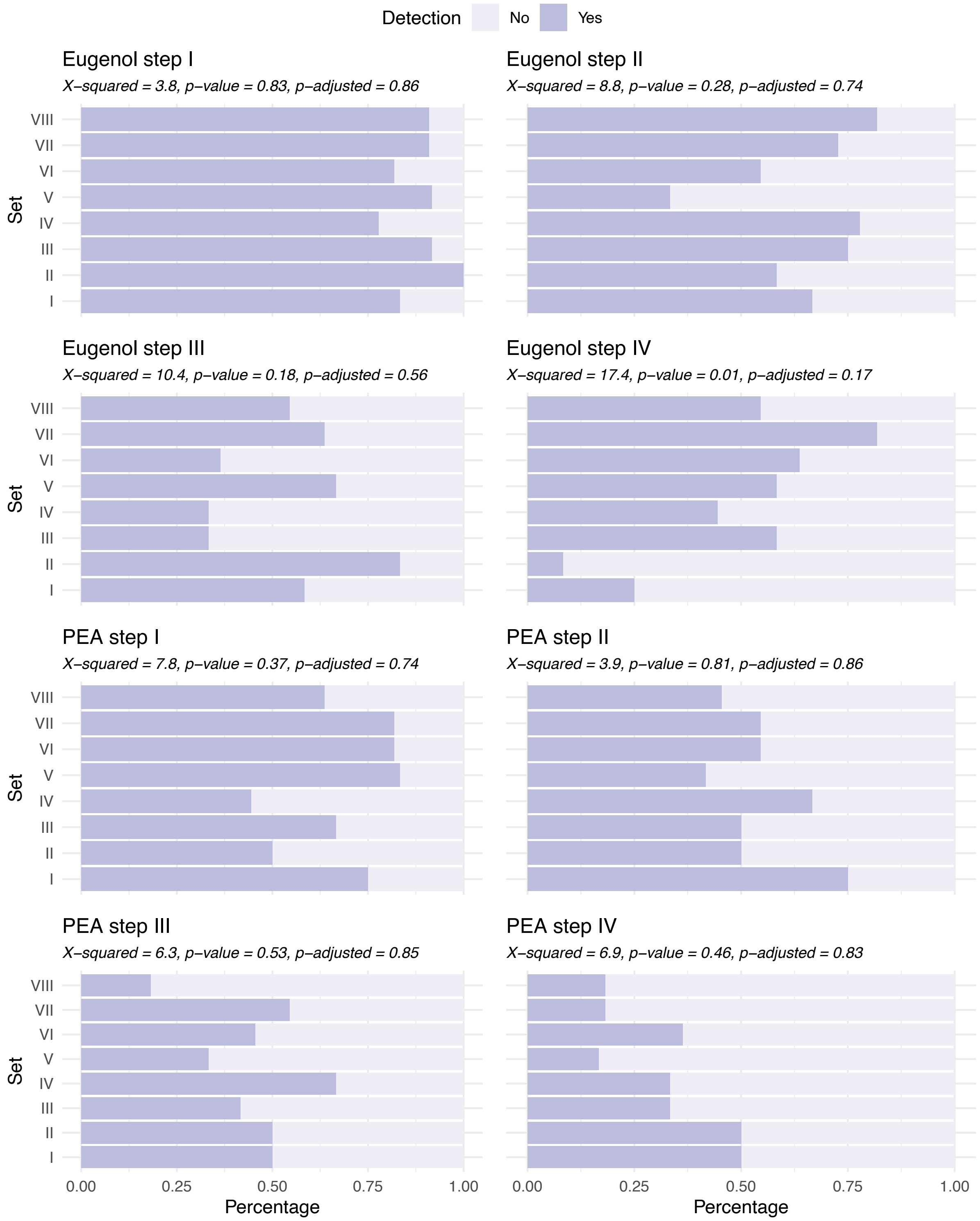

Figure S2. Eugenol and PEA detection rates bar charts for the controls (N = 90) for the second task. Each graph shows the detection rates for one step for the eight odor sets.

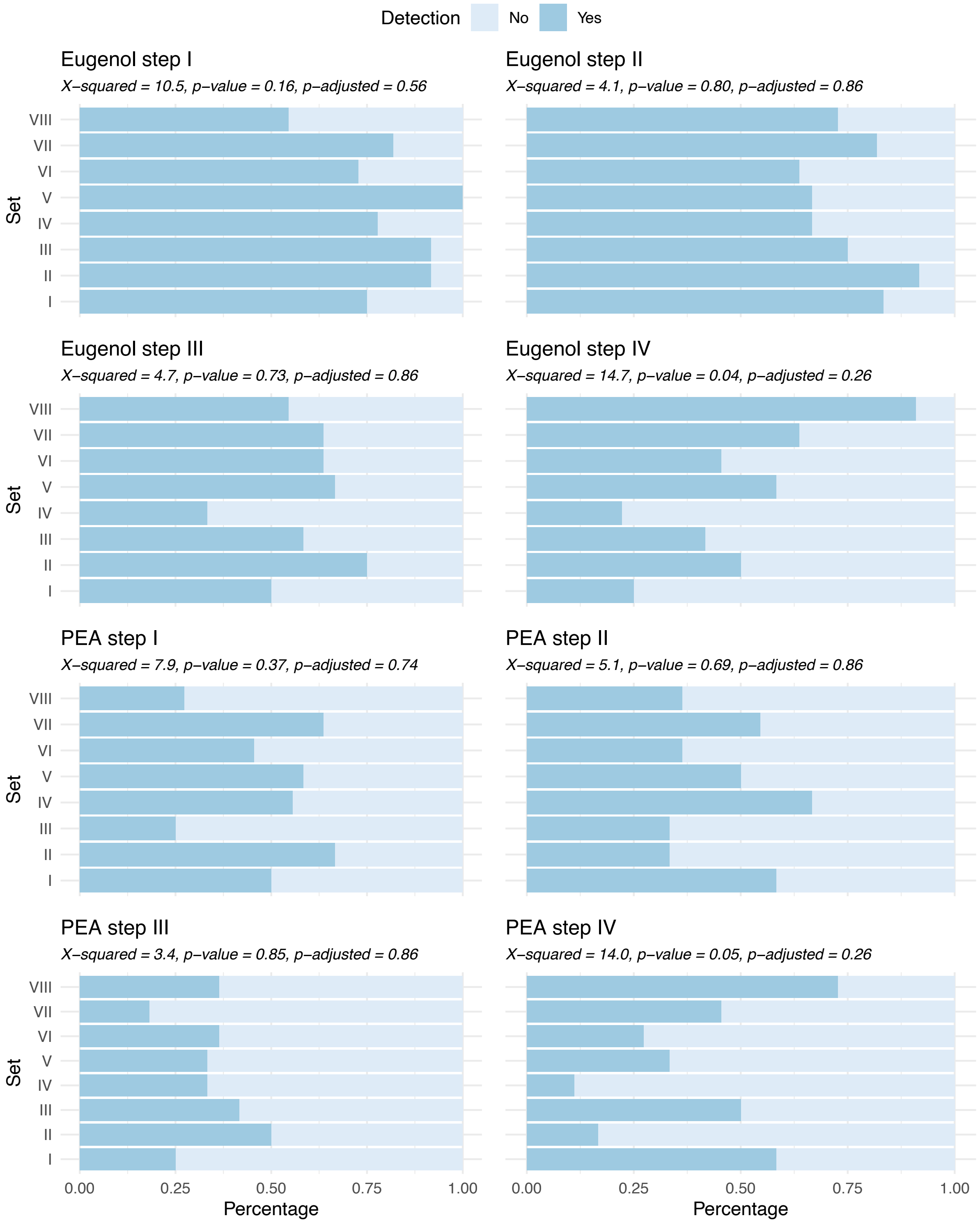

Figure S3. Histograms of the Eugenol detection, PEA detection, and total task scores per task for age- and gender-matched controls (N = 40, above, in pink) and patients (N = 40, below, in blue). Scores were calculated as a sum of successful detection rates for the target odorant for the four steps.

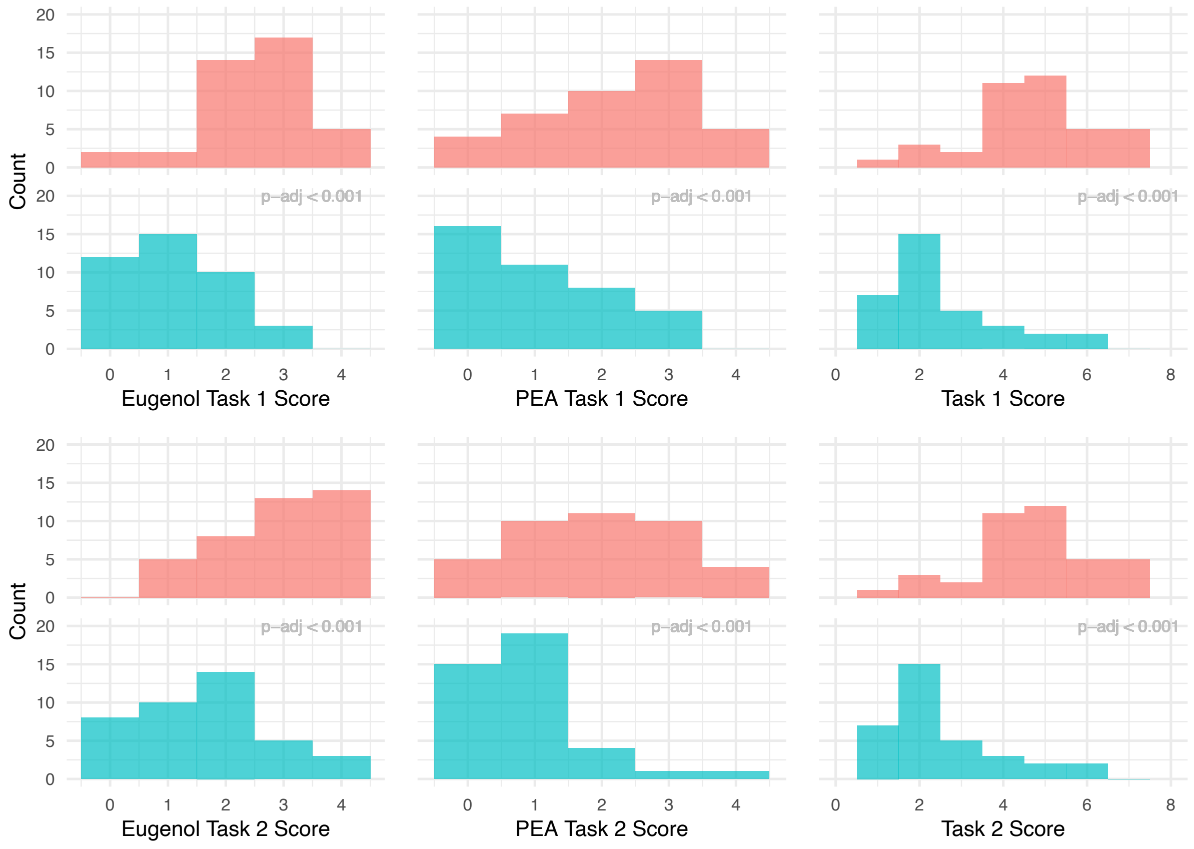

Footnote:

The total scores of Eugenol and PEA detection rates were calculated as the sum of detection rates from 0 (if they could not detect the target in any of the 4 steps) to 4 (if they could detect the target in all 4 steps). The total scores per task were calculated as the sum of detection rates from 0 (if they could not detect the target in any of the four steps for both target odorants) to 8 (if they could detect the target in all four steps for both target odorants).
